# Prioritization of Themes and Research Questions for Health Outcomes in Natural Disasters, Humanitarian Crises or Other Major Healthcare Emergencies

**DOI:** 10.1371/currents.dis.c9c4f4db9887633409182d2864b20c31

**Published:** 2013-10-16

**Authors:** E A P S G: Evidence Aid Priority Setting Group

## Abstract

People making decisions about interventions, actions and strategies for natural disasters, humanitarian crises and other major healthcare emergencies need access to reliable evidence to help ensure that the choices they make are likely to do more good than harm. However, there are many gaps in this evidence base in a wide range of areas. This paper reports a priority setting exercise that was coordinated by Evidence Aid to identify thirty priorities for up-to-date systematic reviews of the effects of interventions, actions and strategies on health outcomes, which would be particularly relevant to those involved in disaster risk reduction, planning response and recovery at an international level. It builds from an ongoing needs assessment that had identified a couple of hundred relevant research questions, which were grouped under 43 themes. Ten of these themes were prioritized by an online survey and the questions attached to these themes were then discussed at a face-to-face meeting, leading to the generation of a list of 30 top priority questions which is presented in this paper. We recognize that a different group of people might have come to different priorities but regard this as an important starting point, and the extensive efforts that were made to be inclusive in gathering opinions should help ensure their wide relevance.

## Background

People making decisions about interventions, actions and strategies for natural disasters, humanitarian crises and other major healthcare emergencies need access to reliable evidence to help ensure that the choices they make are likely to do more good than harm. However, there are many gaps in this evidence base in a wide range of areas.^1^^,^ 2 Furthermore, although some interventions and actions are evidence-based, others are implemented out of convention or on the assumption that the evidence must exist. Furthermore, even if there is some existing evidence, this might come from other settings and not be relevant to the disaster context.

This paper summarizes a priority setting exercise that was coordinated by Evidence Aid to identify 30 priorities for up-to-date systematic reviews of the effects of interventions, actions and strategies on health outcomes that would be particularly relevant to those involved in disaster risk reduction, planning, response and recovery at an international level. It includes the list of these priorities. We used a combination of online surveys and a face-to-face meeting to solicit and rank these priorities, with involvement from low-, middle- and high-income countries, all continents and a range of agencies (see Methods).

Evidence Aid (www.evidenceaid.org) is an international initiative that was established following the tsunami in the Indian Ocean in December 2004.^3^ It uses knowledge from systematic reviews to provide reliable, up-to-date evidence on interventions that might be considered in the context of natural disasters and other major healthcare emergencies. Evidence Aid seeks to highlight which interventions work, which don’t work, which need more research, and which, no matter how well meaning, might be harmful; and to provide this information to agencies and people planning for, or responding to, disasters.

## Objective

The objective of this exercise was to identify approximately 30 high priority research questions under ten themes that could be addressed by systematic reviews in the area of planning for or response to natural disasters, humanitarian crises or other major healthcare emergencies. There was a particular focus on topics of particular relevance to low- and middle-income countries where the health impact of disasters may be greater than in high-income settings.

## Methods

The methods used and the topics considered are outlined below and will be reported more fully in a subsequent report. In summary, this Evidence Aid review priority setting method started with an open, online survey asking humanitarian aid workers and others to provide up to three research questions or areas of uncertainty for which they need research evidence. These suggestions were supplemented at two Evidence Aid conferences and from published literature, before being arranged into themes. A second open, online survey was then conducted to produce the top ten themes, and the associated questions were then prioritized using a nominal group technique with experienced facilitators from the James Lind Alliance,^4^^,^ 5 to arrive at the top 30 priorities for systematic reviews.

Identification of questions and uncertainties

The Evidence Aid online needs assessment survey was opened in July 2011 and is available in Arabic, English, French, German and Spanish.^6^ It has been widely publicised to humanitarian aid workers, agencies, NGOs and others, including those working in low- and middle-income countries through, for example, distribution to a list of people involved in the HINARI initiative which is seeking to improve access to healthcare journals in low- and middle-income countries (www.who.int/hinari/en). The survey remains open, gathering information on the attitudes of people involved in humanitarian responses, disasters and other crises, towards systematic reviews and research in general; priorities for evidence in these settings and preferences for ways to access the information. A preliminary analysis has been published,^7^ and for the purposes of this paper, the focus is on the response to the following three specific questions about systematic reviews and priority setting:

1. In order of decreasing importance what would be your three (or more) priority topics for which you would like evidence in the context of natural disasters or other humanitarian crises?
2. For funders, list any topics for which research evidence would help when making decisions about funding a project
3. For funders , list any topics for which research evidence would help when analysing the reports you receive from recipient agencies

At the time of this prioritization exercise, this first survey had been completed by 101 respondents of whom 83 provided information on the region of the world in which they based. Eleven participants were based in Sub-Saharan Africa (13% of participants who provided their location), 11 (13%) were in Asia, 5 (6%) in the Middle East, 2 (2%) in Eastern Europe and 2 (2%) in South and Central America. The largest proportion of respondents were in Western Europe (23, 28%) with 21 (25%) in North America and 8 (10%) in Australia or New Zealand. Among the 83 first survey respondents who provided information about the organisation they worked for at the time, 34 (41%) respondents worked in international non-governmental organizations (NGOs), 15 (18%) worked in local NGOs; 7 (8%) worked in UN Agencies; 16 (19%) were aid workers working for governments of high-income countries, 4 (5%) were aid workers working for governments in low- and middle-income countries, 6 (7%) worked as academics researchers and 1 (1%) worked for a global organisation.

A list of 216 research questions was compiled from the answers to the relevant sections in the survey, discussions with aid agency research centres and NGOs, ideas from participants and presenters during the Evidence Aid conferences in 2011 and 2012 and published literature (for example, *The Lancet* series on Maternal and Child Health).

Generation of themes

The Evidence Aid team developed the 216 questions further with help from people from relevant agencies and organizations (see Acknowledgements) and grouped the questions into 43 main themes.

Prioritization of themes

An online survey was conducted using SurveyMonkey to prioritize these 43 themes, given that it would be impractical to deal with all of them at the prioritization meeting. The snowballing technique was used for distribution of links to the survey. Information was also distributed using Twitter, Facebook, LinkedIn, the Evidence Aid website, the Active Learning Network for Accountability and Performance in Humanitarian Action (ALNAP) discussion forum, other relevant websites, and a variety of e-mail lists. Recipients were encouraged to complete the survey and to circulate the information about it within their networks. Respondents were asked to select their top ten themes and to rank these from first to tenth. These rankings were then aggregated to generate an overall top ten themes for discussion at the face-to-face prioritization meeting.

Selection of questions

The questions that had been attached to each of the top ten themes were refined, including restricting the topics to those relating to interventions, actions or strategies that might have an impact on health outcomes; and, with help from the Centers for Disease Control and Prevention, they were framed into questions about the effects of the interventions, actions or strategies. Further work reworded the questions so that they would be more suitable for answering through a systematic review.

Prioritization meeting

A table of the questions linked to the ten themes was circulated to participants in advance of the meeting, asking them to prepare for it by ranking the questions within each theme. The meeting was held in London, England on 3-4 June 2013 and was facilitated by people experienced in the James Lind Alliance process (www.lindalliance.org) for reaching consensus in research prioritization.^4^^,^ 5 The participants were divided into small groups to tackle each theme, based on expertise or interest in the relevant topic area, and consensus building discussions were used within the small groups to prioritize the questions, and these priorities were then discussed and agreed with the whole group.

## Results

The online survey of 43 themes was opened by 280 people and 276 of these started to complete the survey, with 233 people submitting their selection of the top ten themes in ranked order. These 233 participants worked for a variety of organizations including international aid agencies, national aid agencies, United Nations agencies, and research centres, as well as people who work independently on issues related to disasters. They came from a varied geographic area: 117 were based in Europe, 45 in the USA, 21 in Asia, 19 in Africa, 12 in the Middle East, 8 in Canada, 7 in South America, and 4 in Australia and New Zealand. The top ten themes that arose from this online survey are shown in Table 1.

**Table 1: Top ten themes chosen from the 43 themes that were ranked by 233 respondents to the online survey:** 

1) Water and sanitation
2) Disaster preparedness
3) Disaster response
4) Nutrition and food security
5) Maternal and child health
6) Co-ordination of humanitarian relief
7) Quality of data/ assessment tools/evaluation/impact
8) Shelter
9) Disaster recovery
10) Mental health

Twenty-eight people from diverse backgrounds relating to disaster management attended the prioritization meeting (Appendix). They were from aid agencies, funders, NGOs, and included independent consultants who had worked in various capacities as well as the facilitators. The meeting led to agreement on a total of 30 prioritized questions for up-to-date systematic reviews (Table 2). It was recognized that further refinements to these questions are likely to take place as they are worked on for systematic reviews, and, as of September 2013, three are already under active discussion with the appropriate groups in The Cochrane Collaboration.^8^

**Table 2: Research questions prioritized for up-to-date systematic reviews at the Evidence Aid prioritization meeting on 3-4 June 2013:** 

What are the effects of point-of-use treatments for water compared to point-of-collection treatments after a disaster or in other humanitarian emergencies?
What are the most effective sanitation and hygiene related personal behaviors after a disaster or in other humanitarian emergencies?
What are the effects of sanitation and hygiene interventions after a disaster or in other humanitarian emergencies?
What are the most effective strategies for waste management in high density emergency settings?
What are the effects and cost effectiveness of investing in disaster preparedness in low- and middle-income countries for reducing excess mortality during a disaster?
What are the effects of community based preparedness on health outcomes after a disaster or in other humanitarian emergencies?
Which human resources and competencies are needed for each phase of disasters (in order to be prepared for future episodes and to have the right people in the right place at the right time)?
What are the effects of different models of co-ordination of humanitarian intervention after a disaster or in other humanitarian emergencies (including financing mechanisms, management mechanisms (e.g. clusters and leadership), information management (e.g. use of new media), communication to and from disaster affected communities?
What are the effects of breastfeeding promotion interventions, including integrated breastfeeding, on breastfeeding rates and duration after a disaster or in other humanitarian emergencies?
What are the effects of emergency feeding programs (including the provision of food, cash and vouchers) after a disaster or in other humanitarian emergencies?
What are the effects of targeted supplementary feeding after a disaster or in other humanitarian emergencies?
How can nutrition or food security information (including probability forecasting) be presented to ensure resource mobilization?
What are the most effective interventions to reduce childhood morbidity and mortality, and improve wellbeing after a disaster or in other humanitarian emergencies?
What are the most effective interventions to reduce maternal morbidity and mortality, and improve wellbeing after a disaster or in other humanitarian emergencies?
What strategies increase the uptake of maternal and neonatal healthcare services after a disaster or in other humanitarian emergencies?
Which are the most effective health and non-health indicators for measuring health outcomes of people affected by a disaster or in other humanitarian emergencies?
What methods can be used to verify the accuracy and reliability of data collected in areas of insecurity, when there is a lack of access to those areas for monitoring purposes?
What is the most effective way to identify the needs of the population affected by a disaster?
What is the impact of the quality of shelter on health outcomes after a disaster or in other humanitarian emergencies?
Which shelter and settlement strategies are optimal under which circumstances (considering progression and long-term consequences) after a disaster or in other humanitarian emergencies?
What are the most effective indicators for measuring the effects of different types of shelter after a disaster or in other humanitarian emergencies?
What is the most effective way to support people who have lost their identity papers (or those who never had papers) to access services after a disaster or in other humanitarian emergencies?
What are the effects of building resilience on “chronic” emergencies (such as famine)?
What is the most effective way of ensuring continuum of care between the response and recovery phases after a disaster or in other humanitarian emergencies?
What are the effects of working closely versus not working closely with government and the private sector in disaster recovery?
What are the effects of starting recovery planning ‘early’ in the response to an emergency?
What are the most effective indicators to monitor and measure mental health and psychosocial support interventions in humanitarian settings?
What are the most effective ways to culturally adapt existing mental health and psychosocial support interventions for use after a disaster or in other humanitarian emergencies?
What are the effects of mental health and psychosocial support interventions after a disaster or in other humanitarian emergencies?
What are the effects of mental health and psychosocial support interventions (e.g. psychological first aid) provided to first responders after a disaster or in other humanitarian emergencies?

## Conclusions

Identifying priorities for research in any area is a demanding and complex task. This is particularly true in the disaster context where the range of evidence needed and the complexity of humanitarian response make it challenging to prioritize key questions that might provide the evidence for decision makers and others making choices about interventions, actions and strategies. However, consensus was reached at this meeting, showing that a group with a diverse knowledge base, responsibilities and experiences could reach agreement on a set of 30 questions to prioritize for up-to-date systematic reviews. We recognise that any process which seeks to draw on such a diverse community is open to potential biases arising from who was invited to take part and who chose to take part. However, the calls for input to both online surveys were wide ranging and inclusive, and the invitations for the workshop were targeted at those with experience or expertise in the ten themes that were to be discussed. We hope that this list of priorities will not only stimulate work to conduct the systematic reviews that will help to answer these questions, but will also encourage debate and action about priorities by others, such as those with a more nationally-orientated focus for disasters.

The next steps for Evidence Aid are for the priorities identified here to be adopted and used to prepare up-to-date systematic reviews of the evidence. This will require the refinement of some of the questions, to improve their suitability as the research question for a systematic review. As with reviews generally, this is likely to be an iterative process, involving key stakeholders in the review and will, ideally, include the prospective registration of the review in PROSPERO and the preparation of a protocol 9 ^,^ 10 .

Furthermore, the reviewers are likely to need to overcome challenges arising from the difficulty of identifying, appraising and synthesizing research that might be spread across hundreds of journals, books, unpublished reports and websites and a lack of uniform reporting of data in the disaster setting. Therefore, we hope that initiatives to improve the accessibility and quality of the underlying research and data, 11 including the development of core outcome sets 12 and templates for data reporting 13 , and acceptance of this list of priorities will support continued progress in this area, and strengthen the evidence base for people making decisions and choices about disaster risk reduction and resilience, planning, response and recovery.

## Appendix 1

### List of people who took part in the prioritization meeting

Claire Allen (Evidence Aid)

Mathias Altmann (Action Contre La Faim)

Stella Anyangwe (Independent consultant)

Grazia M. Caleo (Medecins Sans Frontieres)

Jess Camburn (ELRHA / Save the Children)

Mike Clarke (Evidence Aid)

Rudi Coninx (World Health Organization)

Katherine Cowan (Cowan Associates)

Sally Crowe (Crowe Associates)

Philip du Cros (Medecins Sans Frontieres)

Kate Godden (Nutrition Works)

Brendan Gormley (Independent consultant)

Scott Green (UN Office for the Coordination of Humanitarian Affairs)

Samuel Hauensteinswan (Action Contre La Faim)

Bonnix Kayabu* (Evidence Aid)

Chris Lewis (Department for International Development (DFID))

John Mitchell (ALNAP)

Virginia Murray (Public HealthEngland)

Carlos Navarro-Colorado (Centres for Disease Control)

Cecile Salpeteur (Action Contre La Faim)

David Sanderson (OxfordBrookesUniversity)

Kevin Savage (World Vision International)

Andy Seal (UniversityCollegeLondon)

Emma Sydenham (Cochrane Injuries Group)

Ajay Tripathy (South Asian Cochrane Centre)

Axel van de Veegaete (Red Cross,Brussels)

Mark van Ommeren (World Health Organization)

Vincent Virgo (IFRC and Red Crescent Societies)

Michelle Young (Independent consultant)

*unable to attend the meeting in person
